# Genetic Characterization of Indubrasil Cattle Breed Population

**DOI:** 10.3390/vetsci5040098

**Published:** 2018-12-03

**Authors:** Ricardo Zanella, Luísa V. Lago, Arthur N. da Silva, Fábio Pértille, Nathã S. de Carvalho, João Cláudio do Carmo Panetto, Giovana C. Zanella, Fernanda L. Facioli, Marcos Vinicius G.B. da Silva

**Affiliations:** 1 Faculdade de Agronomia e Medicina Veterinária, Curso de Medicina Veterinária, Universidade de Passo Fundo, BR 285, São José, 99052-900 Passo Fundo, RS, Brazil; lvitorialago96@gmail.com (L.V.L.); arthurnery97@gmail.com (A.N.d.S.); giozanella113@gmail.com (G.C.Z.); 147652@upf.br (F.L.F.); 2 Docente do programa de Pós-Graduação em Bioexperimentação, Universidade de Passo Fundo, BR 285, São José, 99052-900 Passo Fundo, RS, Brazil; 3 Laboratório de Biotecnologia Animal, Departamento de Ciência Animal e Pastagens, Universidade de São Paulo/Escola Superior de Agricultura Luiz de Queiroz, Av. Pádua Dias, 11, 13418-900 Piracicaba, SP, Brazil; fabiopertille@gmail.com; 4 Mestrando do Curso de Zootecnia, Universidade Federal do Rio Grande do Sul/Faculdade de Agronomia, Av. Bento Gonçalves, 7712, 91540-000 Porto Alegre, RS, Brazil; carvalhonatha@hotmail.com; 5 Embrapa Gado de Leite, Rua Eugênio do Nascimento, 610, Dom Bosco, 36038-330 Juiz de Fora, MG, Brazil; joao.panetto@embrapa.br (J.C.d.C.P.); marcos.vb.silva@embrapa.br (M.V.G.B.d.S.)

**Keywords:** Indubrasil, SNPs, inbreeding, pedigree, genetic diversity

## Abstract

The Indubrasil breed was developed in the Brazilian region called Triângulo Mineiro as a result of a cross between zebu cattle. Initially, it was used as a terminal cross and currently it represents approximately 4.45% of all the Brazilian zebu cattle. Studies were conducted to estimate genetic parameters in the Indubrasil using pedigree information, however, until now, no study has been developed using large-scale genomic markers in this breed. Pedigree information are widely used to investigate population parameters; however, they can neglect some estimates when compared to the use of genomic markers. Therefore, the objective of this study was to investigate the population structure and the genetic diversity of Indubrasil cattle using a high-density Single Nucleotide Polymorphism (SNP) panel (Illumina BovineHD BeadChip 700k). Levels of genomic homozygosity were evaluated using three different approaches: Runs of homozygosity (F*_ROH_*), % of homozygosis (F*_SNP_*), and inbreeding coefficient (F*_x_*). Further, Runs of Homozygosity (ROH) segments conserved among the animals were investigated to identify possible regions associated with the breed characteristics. Our results indicate that even the Indubrasil breed having a small effective population size, the levels of homozygosity (F*_ROH_* = 0.046) are still small. This was possibly caused by the cross conducted among different breeds for its development. It suggests no immediate risks associated with loss of genetic variation. This information might be used in breeding programs, for the breed conservation and for the expansion of the Indubrasil breed.

## 1. Introduction

The Indubrasil breed was the first zebuine breed developed in Brazil, in the Triângulo Mineiro region. This breed originated from a cross between the Guzera, Gir, and Nelore breeds, with the objective to maximize the genetic gain of all zebus in only one breed [1]. The breed coat color can vary between white, grey, and red, with thin and short hair and very long ears. The Indubrasil animals have a high robustness and are of a dual-purpose type, for production of both milk and meat [1]. Although more than half of the genealogical records of zebu cattle in Brazil during the 1930s and 1940s have been from Indubrasil animals, the decline of the Indubrasil population in Brasil can be explained by several factors, including a preference for purebred animals, undefined objectives for breed improvement, and unbalanced criterion for animal selection [2]. Between the years of 1925 to 1945, the production of Indubrasil cattle in the country had increased, and in 1946, some of these animals were even exported to the United States for the development of the American Brahman breed (ABCZ). However, currently, the Indubrasil breed represents 4.45% of zebu animals raised in Brazil (ABCZ).

The effective population size (Ne) of the Indubrasil breed has increased from 1956 to 1992, when it reached the highest peak of 292 individuals, followed by a reduction between 1998 and 2000, reaching only 26 individuals [3]. To prevent a reduction of the adaptive value in different populations, it is recommended Ne values between 31 and 250 animals [4]. However, Ne values above 50 are considered to be sufficient to avoid inbreeding depression problems [5], which is considered one of the greatest challenges among breeds with small effective population sizes [6,7]. The inbreeding depression occurs when mating is conducted among related animals, increasing the levels of homozygosity in the offspring and thus the risk that deleterious traits are expressed [8]. However, the system of consanguinity is widely used to fix certain genomic regions associated with characteristics of productive interest [9]. 

The intensification in the use of genomic selection coupled with advanced reproductive technologies have facilitated the rapid spread of animals with higher breeding values. However, the constant use of these animals can drastically reduce the genetic variation among animals, and, therefore, increasing the probability of matings among related individuals [7]. Carneiro et al. (2009) have estimated the Indubrasil inbreeding coefficient (FST) using only the animal pedigree information, and have identified an increase from 0.95% to 7.37% from the second (in 1982) to the sixth (in 2006) generation [3]. This change was possibly caused as a reflex of the reduction in the herd size, verified by the reduction of births per year and the use of fewer animals for reproduction. Schenkel et al. (2002), using pedigree data and breed performance from Indubrasil, Gir, Guzera, Nelore, and Tabapuã have identified that the annual percentage rate of inbreeding after 1980 has increased in almost all the studied breeds, except for Guzera [10]. Between 1992 and 1999, the Indubrasil and Gir breeds presented the highest annual inbreeding rates. In 1999, the Indubrasil breed had 60% of animals mated with close relatives (inbred animals), with an average of inbreeding levels of 7.5% while other zebu breeds presented about 30% of inbreed animals and inbreeding levels below 4% [10]. Estimates of levels of homozygosis using only the pedigree information may underestimate the real levels of endogamy [6,7]. This is possibly caused by failures in the pedigree, lack of complete information, and also factors related to the mendelian segregation [6]. However, the inclusion of genomic information could increase the power of detection of the real levels of homozygosis among individuals [6,7]. Therefore, the objective of this study was to evaluate the levels of genetic diversity in the Indubrasil cattle breed with the use of a high-density SNP panel. 

## 2. Material and Methods

This study was approved by the Embrapa Southeast Livestock Ethical Committee for Animal Use (CEUA-CPPSE) under protocol 02/2009. 

For this study, 38 Indubrasil animals (31 females and 7 males) out of one breeding herd located in the state of Rio Grande do Sul were used. Animals from this herd were bought from different parts of Brazil; therefore, they represent a sample of the Brazilian Indubrasil population. Pedigree information from three generations were obtained by the Brazilian Association of Zebu Breeders public database. Blood samples were individually collected from each animal by the use of vacutainer tubes (with EDTA), and the DNA was extracted using a commercial kit from DNeasy Blood & Tissue Kits (Qiagen, Venlo, Netherlands), following the manufacturer’s recommendations. The DNA quality was evaluated by NanoDrop^®^ ND-2000 spectrophotometer (NanoDrop Technologies Inc. Waltham, MA, USA). DNA samples with 260/280 relation between 1.85–1.95 and 260/230 between 2.0–2.3 were diluted in a final concentration of 500 μg and genotyped at Deoxi Biotectonology Ltd.a using the Illumina’s Bovine700k chip, which interrogates 777,962 SNPs. For the evaluation of levels of homozygosity, only autosome markers were included in the analyses (Table 1). Initially, quality control was performed in the markers excluding SNPs that failed in more than 10% of the samples (call rate < 0.9). After this, we excluded animals that had more than 10% of missing genotypes using PLINK, v1.9 [11]. 

**Figure 1:**
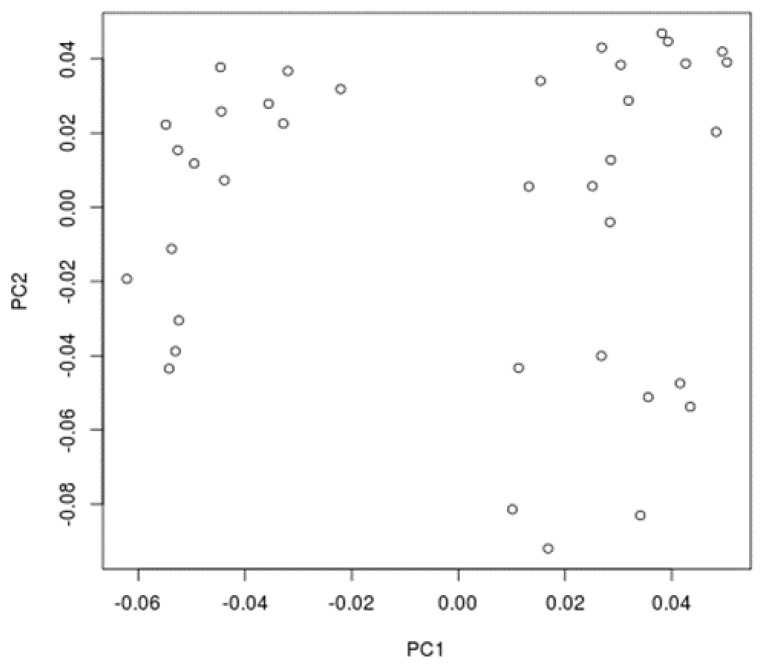
Multi Dimentional Scaling Plot (MDS) graph constructed using post-quality control autosomal markers with r^2^ < 0.2.

**Figure 2:**
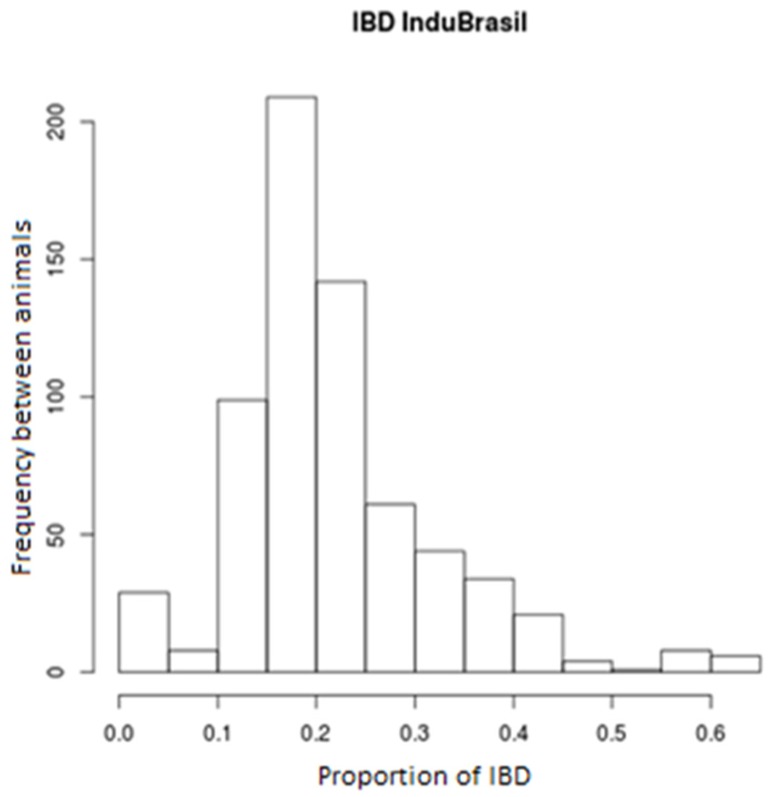
Shared allele’s percentage between animals (IBD).

**Figure 3:**
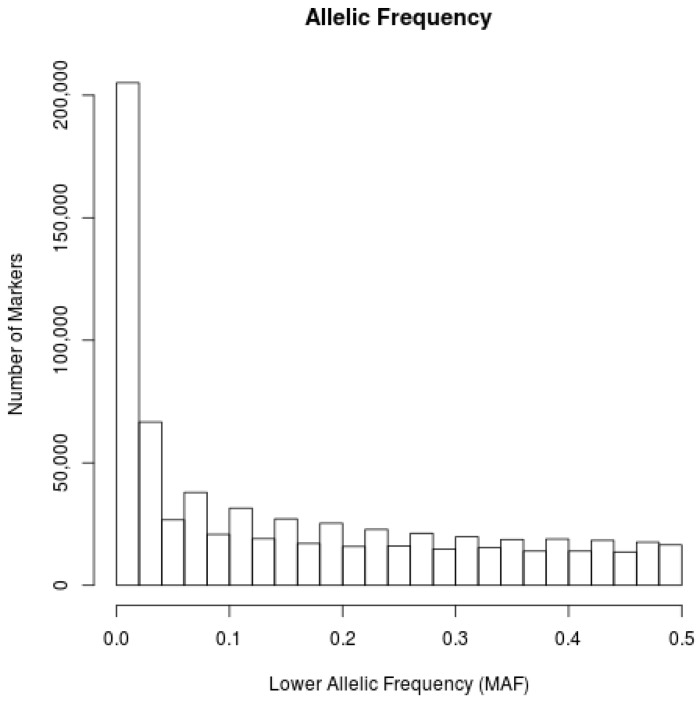
Allelic frequency among the autosomal markers of Indubrasil animals.

In order to verify the genetic similarity among animals used in this study, the identity by state (IBS) levels were computed between all the individuals. Only markers with linkage disequilibrium (LD) levels, r^2^ < 0.2, were used, and the results were plotted in a multidimensional graph (MDS) to facilitate their interpretation (Figure 1). The proportion of genomic segments shared among individuals were estimated through the identity by descent levels (IBD) (Figure 2). The allele frequencies averages were calculated for all the markers in the animals’ autosomal chromosomes (Figure 3).

For the assessment of genomic homozygosis levels, three methodologies were used:

(1) The percentage of homozygous genotypes in relation to the total number of genotypes (F*_SNP_*).
(2) The inbreeding coefficient (F*_x_*) using the frequency of the observed homozygous genotypes (HO) in relation to the expected homozygous genotypes (HE): F*_x_* = [(HO − HE)/(HE)]).
(3) Runs of homozygosis (ROH). For the verification of F*_ROH_*, the ROH were detected using the PLINK program with a sliding window of 50 SNPs with minimum length of 1000 kb, with a possibility to have 1 heterozygous SNP and 1 missing SNP within each window. For this, we used the following formula:
  F*_ROH_* = Σ_k_ Length (*ROH*_k_)/L

where “k” is the number of ROHs identified in each individual multiplied by the segment size average in Kb, and L is the total size of the cattle genome (2,612,820 Kb) [6].

The linkage disequilibrium levels (r^2^) and the number of regions of homozygosis (ROH) were estimated by chromosomes (Table 2) in PLINK v1.9. Regions of homozygosis shared in more than 50% of the individuals were identified using PLINK v1.9, and investigated for the identification of genes fixed in the Indubrasil breed using the UCSC Genome Browser [12] (Table 3).

## 3. Results

After the quality control, 735,293 SNPs were identified to be located on the autosomal chromosomes. From these, 13,699 SNPs were removed because they failed in more than 10% of the samples (call rate < 0.9). After quality control, 721,594 autosomal SNPs and 37 animals were retained, 30 (*n* = 30) females and seven (*n* = 7) males with an average genotyping call rate of 99.26%. One female was excluded from the analysis for having more than 10% of missing genotypes. 

The IBS levels estimated among animals with the use of markers with r^2^ < 0.2 (*n* = 45,334 SNPs) indicates a genomic heterogeneity among the animals studied (Figure 1). The average IBD levels among all the animals was 0.218 ± 0.104, ranging from 0 and 0.623. This suggests a degree of variation among the sampled animals. In our study, we had animals related with each other as well as animals without any genetic relationship (IBD = 0) (Figure 2). 

**Figure 4:**
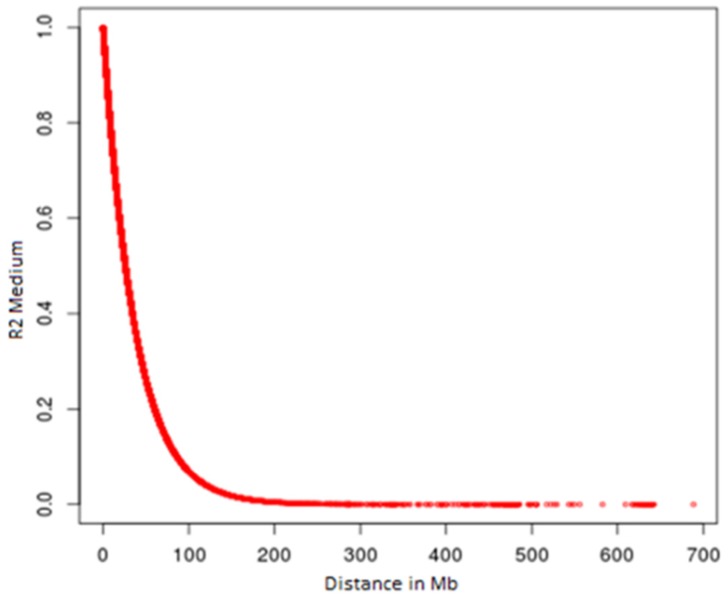
Break in r^2^ levels with the increase distances between markers.

In relation to the allelic frequencies of all 735,293 markers analyzed, an average frequency of 0.161 was identified among all the markers. This indicates a moderate genetic variation between autosomal markers of Indubrasil cattle in this study (Figure 3). It was observed that as the distance between the markers increased, the values of linkage disequilibrium decreased, with distances above 70 Kb, the average r² was smaller than 0.20 (Figure 4).

We also observed a high correlation r = 0.74, between the number of markers and regions of ROH in chromosomes, being that the chromosome 5 (*BTA5*) was the one with the largest amount of regions in homozygosity shared among the animals (Table 2). Segments of the *BTA6* were the only shared with more than 50% of the individuals, being that the region, 6:38,698.886–39.581,936 bp, was shared with 86% of the animals in our sample. This region contains the genes: *DCAF16*, *NCAPG*, and *LCORL* (Table 3).

The levels of genomics homozygosis calculated by the average percentage of homozygous genotypes (F*_SNP_*) in this population was 0.713 ± 0.02 (min = 0.673, max = 0.761) (Table 4) being F*_SNP_* = 0.718 for males and F*_SNP_* = 0.712 for females. The estimated coefficient of inbreeding (F*_x_*) was calculated by the excess of homozygous markers in an individual, through the PLINK, it was found an average of −0.032 ± 0.075, minimum value of −0.181, maximum value of 0.135 (Table 3). Males showed an F*_x_* of −0.020 and females an F*_x_* of −0.039. Using the runs of homozygosis (F*_ROH_*), we have identified an average of F*_ROH_* = 0.04 ± 0.035, minimum of 0.0126 and maximum of 0.18 (Table 4). In males, the F*_ROH_* was 0.040 and in females, was 0.047. A strong positive correlation, r > 0.752, was identified between the different methods of genomic inbreeding estimation, indicating a consistency in the evaluation of genomic inbreeding by different methodologies in this population (Table 5).

In the population studied, 5.4% of the animals presented recent inbreeding events with an average segments size between 3.5 and 4.0 Mb. Meanwhile, 18.93% of the animals presented ROH segments with average sizes between 2.5 and 3.5 MBs, and lastly, 75.67% presented ROH segments below 2.5 Mb, indicating older inbreeding mating.

## 4. Discussion

The estimation of the levels of homozygosity reveals important information about the genetic diversity of animals from different populations. In addition to that, this information can be used as a strategy to conduct mating in different selection programs [7]. Maintaining low levels of endogamy and generating high levels of genetic variation is a challenge facing the livestock sector, mainly with the intensification of the production system.

The increased levels of homozygosis may reflect what has occurred in the past or in a recent process with the intensification of the selection pressure among animals [13]. While the coefficient of traditional inbreeding (FST) reflects the inbreeding in later generations, the coefficients based on ROH are able to detect both the recent and more distant inbreeding [14,15]. The F*_ROH_* is based on the segments of homozygosis of each animal, being a more specific evaluation and accurate than the estimates performed with pedigree data, which sometimes can be incomplete and contain identification errors [16,17]. The genomic regions under strong selection pressure have shown a high number of events of inbreeding [18]. Long ROH indicate recent events of biparental inbreeding while short ROH indicate more ancient endogamy [7,17]. Zavarez (2015), investigating the levels of genomic homozygosis in Nelore breed animals genotyped with an Illumina SNP HD chip, have identified average values of ROH length of 1.26 Mb, varying from 0.5 to 70.91 [19]. Segments larger than 10 Mb are marks of inbred matings that have occurred in the last five generations [20]. From the Indubrasil animals genotyped in this study, 81% had at least one fragment exceeding 10 Mb. This may be a reflection of the cumulative matings performed in the past due to intensification in the animals’ selection.

Vercesi Filho et al. (2002) studied the Indubrasil population evolution using the Wright’s inbreeding coefficient (FST) and verified an increase between 1938 and 1998 from 1% to 3.4% [21]. In our study, the inbreeding estimates, with the use of runs of homozygosis, presented an average value of 4.61% of the population studied and a homozygosis percentage average of 71.3%. Our results were higher than those found by Vercesi Filho et al. (2002), suggesting a possible refinement of the race, and consequently fixation of the genomic segments associated with breed-specific traits, which have already been determined, or an underestimation of inbreeding values by using only the pedigree.

Using Wright’s Inbreeding Coefficient (1931) [22], Oliveira et al. (1999) evaluated a herd of Guzera cattle, finding a variation of 0 to 25% for males and 0 to 31% for females, with an average of F*_x_* = 1.08% and 1.36% [23]. The low level of inbreeding estimates observed in the herd can be a consequence of the small number of generations analyzed (~3) in addition to the reproductive processes conducted to avoid in the bovine breed. These data are similar to those found by Muniz et al. (2012), that assessing the population structure of bovine’s breed, Gir Mocha, estimated the levels of endogamy evaluated by the pedigree of animals [24]. The result was zero from 1954 to 1984, and between the years of 1985 to 1997, were found values of F*_x_* below 1%, indicating little loss of genetic variability.

Common regions of ROH shared among individuals are known as homozygous islands [19]. In accordance with Nothnagel et al. (2009), these regions in humans, when they are present in more than 50% of the individuals of a population, can indicate a strong selection occurrence [25].

An RNA sequencing study in the Qinchuan chinese cattle showed different levels of *NCAPG* gene expression, located in *BTA6* and related with the development of the longissimus dorsi muscle [26]. In the same study, using qPCR, two adjacent genes to *NCAPG* were differentially expressed. These two genes, *LCORL* and *DCAF16*, have higher levels of expression in fetal muscle, suggesting their involvement in muscular development. In addition, the *HCAP-G (NCAPG)* and *LCORL* genes were suggested to be associated with production traits in beef cattle. These traits are: Weight of the carcass [27], calving ease [28,29], height in adulthood, survival of the calf at birth [30], feed intake, weight gain, rib eye area, and subcutaneous fat [31]. This region was also associated with the total proportion of bones, birth weight, birth length, dystocia, and fetal death. In the present study, it was observed that 86% of the evaluated Indubrasil animals shared a region on chromosome 6 (6:38,698,886–39,581,936 bp), which anchors the same genes discussed above (Table 2).

Studies with European breeds found QTLs in this chromosome 6 region associated with productivity, fat, and milk protein [13]. Silva (2010), in a study using Gir breed animals, proved that even with the great genetic diversity observed between taurine and zebu breeds, the QTL found in *BTA6* related to milk production traits were also identified in the zebu breed [32]. This suggests that a possible selection is being carried out to implement the milk production in the Indubrasil cattle population investigated here. Selection for certain traits favors the fixation of these regions in selected populations. This information is confirmed by the milk production aptitude and longevity of the Indubrasil (ACGZ- Associação dos Criadores Gaúchos de Zebu (http://www.acgz.com.br/)).

In general, our results suggest that the inclusion of genomic data is of extreme importance to avoid possible problems related with the inbreeding depression, caused by the reduction of genetic variability in animals. In this way, it can be used as a criterion of selection and crossing of individuals in order to standardize the breed and to implement the selected traits in shorter time.

## 5. Conclusions

In this work, parameters related to inbreeding in a sample population of Indubrasil animals were evaluated with the use of a high-density SNP panel. Animals in the present study have demonstrated low levels of genomics endogamy, not evidencing immediate risks related with inbreeding depression, possibly due to the previous crosses among different breeds made before the origin of the Indubrasil.

## Figures and Tables

**Table 1:** Number of markers per chromosome.

Chromosome (BTA)	Markers (SNPs)
1	46,495
2	40,056
3	35,579
4	34,980
5	34,842
6	35,519
7	33,168
8	33,529
9	31,060
10	30,449
11	32,015
12	26,127
13	23,594
14	24,780
15	24,755
16	24,178
17	22,266
18	19,386
19	18,908
20	21,490
21	21,175
22	18,034
23	15,215
24	18,620
25	12,931
26	15,242
27	13,152
28	13,038
29	14,710
Total	735,293

**Table 2:** Homozygous regions per chromosome.

Chromosome (BTA)	Number of Shared Regions (ROH)
1	43
2	33
3	23
4	19
5	53
6	51
7	28
8	25
9	20
10	34
11	28
12	32
13	28
14	22
15	17
16	19
17	17
18	23
19	20
20	19
21	21
22	20
23	7
24	19
25	15
26	17
27	11
28	5
29	18
Total	687

**Table 3:** Regions of homozygosity shared with more than 50% of the animals.

% of Animals	Cromosome (BTA)	Initial Position (bp)	Final Position (bp)	Genes
51%	6	34,496,622	34,671,490	-None-
54%	6	36,717,524	36,738,110	-None-
59%	6	35,211,888	35,992,229	*CCSER1*
62%	6	37,380,548	38,188,436	*FAM13A*, *HERC3*, *NAP1L5*, *PIGY*, *PYURF*, *HERC5*, *HERC6*, *PPM1K*, *ABCG2*, *PKD2*, *SPP1*
65%	6	40,186,267	41,452,236	*SLIT2*
86%	6	38,698,886	39,581,936	*DCAF16*, *NCAPG*, *LCORL*

**Table 4:** Estimated levels of inbreeding.

	Average	Standard Deviation	Minimun	Maximum
F*_x_*	−0.032	0.075	−0.181	0.135
F*_ROH_*	0.046	0.035	0.012	0.180
F*_SNP_*	0.713	0.020	0.673	0.761

**Table 5:** Correlations between the different methods of inbreeding estimates.

r	F*_x_*	F*_ROH_*	F*_SNP_*
F*_x_*	1	0.752	0.999
F*_ROH_*	0.752	1	0.752
F*_SNP_*	0.999	0.752	1
